# A Plan for the Ethical Dental Education of the Public

**Published:** 1921-09

**Authors:** W. Linford Smith


### A PLAN FOR THE ETHICAL DENTAL EDUCATION OF THE PUBLIC

BY W. LINFORD SMITH

One of the innumerable quotations which could be made to demonstrate the interest of the profession in the ethical dental education of the public is the statement made by Dr. A. E. Webster, Dean of the Royal College of Dental Surgery of Toronto.

In a paper read at Washington in April of 1920, he said: "Every dental society and every dental organization since its inception has recognized the need of public dental education, and has appointed committee after committee these fifty years to prepare articles for the press and for general distribution, with the result that now and then something is done, but without any continuous or persistent policy being followed. Sporadic advertising is useless and so is sporadic dental education of the public."

That Dr. Webster is absolutely correct as to the attitude of organized dentistry on this subject is proven by the interest which has been taken by members of the profession in the distribution of the syndicated "Your Teeth" articles which, at present, are appearing weekly in no less than 373 newspapers throughout the United States and Canada.

These articles are written by Dr. Rea Proctor McGee, and through the pages of the magazine which he edits, the service is offered without charge to any newspaper willing to carry the matter, provided the request is made by a local organization of dentists.

Readers of the magazine are doubtless familiar with the character of the articles and the plan of distribution.

The 373 newspapers in which these articles appear are read by 3,817,860 people.

If the layman is benefited by reading in the pages of his newspaper authoritative information on the subject of dentistry, it is perfectly obvious that he would derive very much greater benefits if means could be devised for supplementing the newspaper campaign by a series of cards containing similar matter, mailed to his home address.

Such a direct appeal would be the ideal way of accomplishing the desired result, but the cost involved would be impossible to finance by any ordinary means.

The object of this paper therefore is to submit for your consideration (a plan whereby direct dental education of the public may be accomplished in an ethical manner), and at the same time have the burden of cost so widely distributed that it will not be felt by any individual.

To this end it is proposed to print on postal cards, a series of twelve messages of approximately 150 words each, and to arrange for their widest possible distribution through the co-operation of the individual dentist in his local community.

To safeguard the interests of the profession it is desired that a Committee or Board of Censors be appointed by the National Dental Association or its Oral Hygiene Section, and that permission be granted to print on each card the fact that the subject matter is endorsed or approved by some such eminent authority.

A series of twelve articles have already been prepared, and if the co-operation we seek is granted, they will be submitted to the proper authority for approval.

Should the text of the cards not meet with approval, or should any exception be taken to words or phrases employed, the reading matter will either be rewritten in its entirety, or modified in any way that may be suggested. The cards will bear no form of advertising matter of any sort or description.

The organization and facilities for distribution possessed by the American Dental Trade Association will then be put in operation for securing from the individual dentist lists of persons who, in his opinion, will be benefited by reading strictly ethical information as to what dentistry stands for.

The suggestion will be offered that these lists should be compiled from local organizations—civic, fraternal, religious, political, educational, etc., or in smaller communities, the cards could be mailed to the home address of each individual taxpayer.

When the lists will have been received, the names they contain will be transferred to stencils, and machinery will be installed at a Central Bureau, where a sufficient clerical force will be maintained to keep the lists alive and to handle the addressing and mailing of the cards.

To assist in covering the cost of the campaign, the service will be offered to individual dentists at $18 per annum, for the entire series of twelve, (which is $1.50 per month), for each 100 cards.

Twelve dollars of this amount will be required for postage alone, and it is hoped that the balance will defray the cost of printing, addressing, mailing the cards, etc. Whatever deficit results, those whom I represent will pay the bill.

No person, firm or group of men either hope or except to derive any profit from the proposition. No salaries will be paid to those in authority, and no expense will be involved, aside from the actual cost of maintaining the service and details incident to putting the plan in operation.

If the co-operation of the National Dental Association is granted an intensive eight weeks' drive will be inaugurated on the first of October of this year which I venture the prediction will result in the distribution of a minimum of a million cards per month beginning January 1st.

This will mean delivery to the homes of at least that many American citizens, the message of dentistry and what it stands for.

And one million circulation of this character would not mean circulation in the sense used by publishers; it would mean a minimum of one million, and a maximum of five million actual readers which, it is safe to say, are half again as many people as there are today in North America who really recognize in dentistry a vital factor of physical welfare.

In submitting this plan for your consideration, I am dealing with a definite and a concrete proposition.

The fact and figures quoted are in no sense merely tentative estimates as to what can be done.

Organized dentistry for years has sought a means of educating the public, but has been deterred by the enormous expense involved.

There is no question as to the desire of organized dentistry that the public should be educated on this vital topic.

So then, when it is proposed that this be accomplished and that the National Dental Association co-operate and yet be relieved of the enormous amount of work involved in making the plan effective, it is hoped that the proposal will receive favorable consideration at your hands.

It is impossible in the brief space of time allotted to discuss details regarding the means which have already been devised for such a stupendous undertaking.

But every detail of this proposal has been worked out with the utmost care and every angle will be discussed frankly with any Committee which may be appointed to co-operate with us.

The plan is feasible and workable, and its possibilities have been under rather than overestimated.

The subject of this paper is "A Plan for the Ethical Dental Education of the Public."

The two questions respectfully submitted for your consideration are:

First, does the National Dental Association desire that the public be educated?

Second, if so, is the National Dental Association under proper safeguards, willing to approve a plan which is rendered practical by utilizing the organization and the facilities of those whom I represent?

